# Peak alpha frequency as an objective biomarker for cognitive assessment in post-stroke cognitive impairment

**DOI:** 10.3389/fnagi.2025.1639970

**Published:** 2025-10-23

**Authors:** Yuqing Zhao, Haoran Shi, Weicheng Kong, Xinyang Wang, Wei Wei, Zengtu Zhan, Xiehua Xue

**Affiliations:** ^1^College of Rehabilitation Medicine, Fujian University of Traditional Chinese Medicine, Fuzhou, China; ^2^The Affiliated Rehabilitation Hospital, Fujian University of Traditional Chinese Medicine, Fuzhou, China; ^3^Fujian Provincial Rehabilitation Industrial Institution, Fujian Provincial Key Laboratory of Rehabilitation Technology, Fujian Key Laboratory of Cognitive Rehabilitation, Fuzhou, China

**Keywords:** poststroke cognitive impairment, PAF, Montreal Cognitive Assessment, EEG, PSD

## Abstract

**Objective:**

To investigate regional associations between peak alpha frequency (PAF) and poststroke cognitive impairment (PSCI) and evaluate PAF as an objective biomarker for cognitive assessment in PSCI.

**Methods:**

A cross-sectional study compared 103 participants [PSCI, poststroke non-impaired (PSN), and healthy controls]. Cognitive function was assessed using MoCA scores. PAF characteristics were analyzed across brain regions via EEG, with logistic regression and Random Forest identifying key predictors. We aimed to evaluate whether PAF can be an effective indicator of cognitive status in PSCI.

**Results:**

The Kruskal-Wallis test with *post hoc* Bonferroni correction revealed that PSCI exhibited significantly lower PAF compared to HC across all major brain regions (frontal, temporal, central, and parieto-occipital; all *P* < 0.05). Compared to PSN, the PSCI group showed significantly reduced PAF at specific electrodes (F3, F4, F7, T3, T6, Fz; *P* < 0.05). Spearman correlation analysis demonstrated that PAF at multiple leads was positively correlated with MoCA scores across all subjects. Notably, after FDR correction, only T3PAF and T4PAF remained significantly negatively correlated with MoCA in all subjects (*q* < 0.05). Binary logistic regression identified T4PAF as the most discriminative predictor for distinguishing PSCI from HC (OR = 2.525). Random Forest analysis corroborated these findings, identifying F7PAF, O2PAF, T3PAF, and T4PAF as the most important predictors. Both models demonstrated excellent discriminatory power, with AUCs of 0.761 (logistic regression) and 0.773 (Random Forest), indicating robust performance of EEG-based biomarkers for PSCI detection.

**Conclusion:**

Peak alpha frequency serves as a robust electrophysiological biomarker for PSCI. Multi-region PAF analysis enhances diagnostic precision for poststroke cognitive decline.

## Introduction

Poststroke cognitive impairment (PSCI) has a spectrum of severity ranging from mild to severe and affects up to 60% of stroke survivors within the first year after stroke (Douiri et al., 2013; Jacquin et al., 2014; Lo et al., 2019). It has been reported that 20% of mild PSCI patients who receive early intervention can recover completely within 2 years (Rasquin et al., 2005).

The assessment of PSCI is mostly based on subjective scales, Mini-Mental State Examination (Burton and Tyson, 2015; Stolwyk et al., 2014) and the Montreal Cognitive Assessment (Burton and Tyson, 2015; Hachinski et al., 2006) have been the most widely studied cognitive screening instruments. But most screening instruments were not developed to identify the heterogeneous presentation of poststroke cognitive deficit and might miss subtle (yet impactful) poststroke cognitive changes (El Husseini et al., 2023). Furthermore, stroke-related impairments may render standard cognitive screening tools inadequate, such as motor weakness, unilateral neglect, and aphasia, as well as demographic factors such as education, language, or culture (El Husseini et al., 2023). There is a growing consensus supporting the incorporation of objective, neurophysiological markers that reflect biological mechanisms of disease to improve PSCI assessment (Biesbroek and Biessels, 2023). Electroencephalography (EEG) offers a promising avenue in this regard. EEG captures electrophysiological brain activity and has the advantage of being applicable across all patient populations.

Previous work suggested that individual performance in cognitive can be predicted by resting state oscillatory neuronal activity (Clements et al., 2021; Klimesch, 1997; Mahjoory et al., 2019). The electrical signal generated by the activity of brain neurons can indirectly reflect the pathological and physiological information of the subject. EEG mainly generates different waveforms according to spontaneous electrophysiological activity signals generated by the brain, it can reflect changes in brain function and activity (Müller-Putz, 2020). In particular, alpha-band activity has emerged as a central focus due to its role as the dominant resting-state rhythm and its widespread distribution across cortical networks. Alpha oscillations are believed to constitute a structural and functional foundation for cognitive control (Haegens et al., 2015; Sadaghiani and Kleinschmidt, 2016). Among various EEG metrics, the peak alpha frequency (PAF)–the frequency within the alpha band exhibiting maximal power (Finley et al., 2024; Keitel et al., 2019; Ramsay et al., 2021) –has proven to be a stable and reliable neurophysiological trait associated with cognitive performance (Perez et al., 2024). In healthy adults, higher PAF is correlated with better cognitive abilities (Grandy et al., 2013; Finley et al., 2024). Notably, PAF has also demonstrated predictive value in stroke contexts, with one study reporting 74.2% accuracy in predicting cognitive outcomes after stroke (Schleiger et al., 2017). These findings position PAF as a promising electrophysiological marker capable of complementing existing clinical evaluations.

Research indicates that inter-individual differences in PAF demonstrate the characteristics of a stable neurophysiological trait. This robust index appears to be unaltered by the presence of subjective memory complaints (Poland et al., 2021). Longitudinal studies further indicate that PAF may serve as a prognostic marker for cognitive decline from midlife to older age (Finley et al., 2024). A review in the same year also confirmed that higher PAF was associated with higher intelligence, executive function, and general cognitive performance scores. A review (Chino et al., n.d.) suggests that a higher PAF is linked with a higher score in intelligence, executive function, and general cognitive performance and could be considered an optimal, and easy-to-assess, electrophysiological marker of cognitive health in older adults. Notably, PAF has also demonstrated predictive value in stroke contexts, with one study reporting 74.2% accuracy in predicting cognitive outcomes after stroke (Schleiger et al., 2017). Given its predictive value for such a broad range of cognitive abilities, some researchers have even proposed that PAF may serve as a marker of general intelligence (Grandy et al., 2013). These findings position PAF as a promising electrophysiological marker capable of complementing existing clinical evaluations.

We aim to investigate PAF as an electrophysiological biomarker for PSCI, with the goal of identifying effective and sensitive indicators that can complement standard clinical assessments. Our specific objectives are to: (1) quantitatively evaluate PAF parameters in individuals with PSCI, and (2) establish cut-off values for these alpha-band activity metrics that demonstrate high screening accuracy for cognitive impairment. To our knowledge, this is the first study to examine PAF specifically in PSCI, offering novel insights into neurophysiological markers relevant to cognitive rehabilitation after stroke.

## Materials and methods

Ethical approval for this study was granted by the Ethics Committee of the Rehabilitation Hospital affiliated with Fujian University of Chinese Medicine (2023YJS-003-01). The research was carried out in compliance with the World Medical Association Declaration of Helsinki. Written informed consent was obtained from all patients prior to their inclusion in the study.

### Study design and population

A total of 41 PSCI patients,24 PSN (Post-Stroke Cognitively Normal) patients were recruited from the Rehabilitation Hospital affiliated with Fujian University of Chinese Medicine (Fuzhou, China); at the same time, 38 healthy controls matched by age were included (Table 1). The diagnosis of PSCI comprises the following criteria: (i) a definitive stroke diagnosis supported by clinical or imaging evidence, encompassing hemorrhagic stroke, and ischemic stroke; (ii) the presence of cognitive impairment, as reported by patients or informed observers or as assessed by experienced clinicians and confirmed by neuropsychological evidence of functional impairment in multiple cognitive areas or evidence of significant cognitive decline compared to previous levels; and (iii) a temporal relationship between the stroke event and the onset of cognitive impairment, with symptoms persisting for 3–6 months poststroke. All subjects completed the neuropathology scale and EEG examination within 7 days after enrollment. All the subjects volunteered to participate in the study and provided written informed consent.

**TABLE 1:** Clinical characteristics of PSCI and healthy group.

Variable	PSCI group (*n* = 41)	PSN group (*n* = 24)	Healthy control (*n* = 38)	Statistical value	*P* value
Sex (Male/Female)	30/11	18/6	25/13	*X*^2^ = 0.349	0.706
Age (years)	61.30 ± 10.27	59.58 ± 8.50	63.21 ± 5.88	*F* = 1.382	0.256
MoCA score	18.34 ± 3.42	26.38 ± 0.92	26.21 ± 1.34	*F* = 141.46	<0.0001*
Hemisphere (Right/Left)	25/16	45855	–	*X*^2^ = 2.828	0.43

Continuous variables were presented as mean ± standard deviation, categorical variables were presented as frequencies.

*P < 0.05, indicating that there is a significant statistical difference in the MoCA score between groups.

**TABLE 2:** Regression results for the relationship between alpha activity and PSCI.

Indices	β coefficient	Wald *X*^2^	OR	*P*	95% CI
C3PAF	0.611	7.598	1.842	0.006	1.193, 2.844
P4PAF	0.732	8.902	2.080	0.003	1.286, 3.364
O1PAF	0.457	3.832	1.579	0.05	0.999, 2.494
O2PAF	0.897	10.000	2.451	0.002	1.406, 4.272
F8PAF	0.551	5.124	1.735	0.024	1.077, 2.796
T3PAF	0.767	7.867	2.154	0.005	1.260, 3.681
**T4PAF**	**0.926**	**11.791**	**2.525**	**0.001**	**1.488, 4.284**
T5PAF	0.599	6.781	1.820	0.009	1.160,2.856
T6PAF	1.003	11.164	2.726	0.001	1.514, 4.910
CZPAF	0.551	5.124	1.735	0.024	1.077, 2.796

Employed forward stepwise regression to identify independent variables. The culminating model comprised of T4PAF as its predictive components (Bold). Data presented as β coefficients. The dependent variables are PSCI or not, whereas independent variables are alpha activity indices. This means that the β coefficients just indicate in which direction (positive or negative) and how strong the associations are. PAF, peak alpha frequency; OR, odds ratio; 95% CI, confidence interval.

We also employed the Random Forest (RF) algorithm for both feature selection and predictive model construction. In our model, the error rate stabilized when ntree was set to 1000. The RF model can quantify the influence of each independent variable on the dependent variable and calculate importance scores (Supplementary Figure 2). The feature set for this study comprised PAF values from all 19 channels. Thus, each participant was represented by a 19-dimensional feature vector. These feature vectors, along with their class labels (PSCI or HC), formed the final dataset. A Random Forest classifier was employed to discriminate between PSCI patients and HCs. The model was implemented using the Scikit-learn library in Python. The dataset was randomly split into a training set and a hold-out test set with a ratio of 4:1. The model’s performance was evaluated on the test set using the Area Under the Receiver Operating Characteristic Curve (AUC-ROC), accuracy, sensitivity, and specificity (Supplementary Figure 3). Our study developed a Random Forest model that showed promising discriminative ability (AUC = 0.818) (Supplementary Figure 2) on an independent test set for classifying stroke patients. However, we acknowledge the limitations raised by the internal cross-validation. The considerable variability in the cross-validation AUC scores (mean: 0.689 ± 0.230) suggests that the model’s performance is not yet fully stable, likely due to the constrained sample size of our cohort.

**FIGURE 6:**
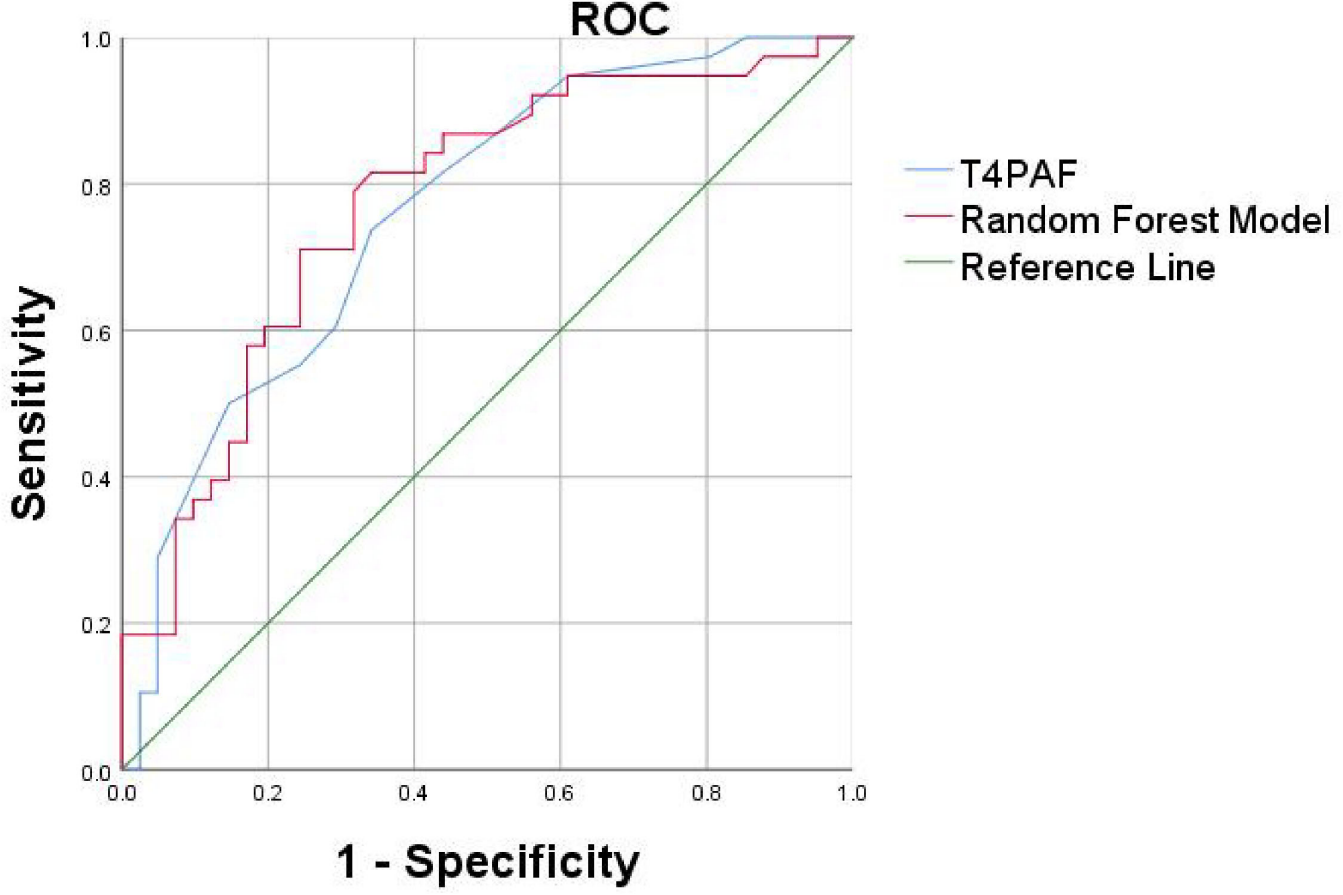
Receiver operating characteristic curves based on the Random Forest (RF) and logistic regression method of the differential PAF between PSCI and HC. The AUC was 0.761 for logistic regression model (blue curve for T4PAF), 0.773 for random forest model (red curve for O2, T3, T4, T5PAF combined). PAF, peak alpha frequency; ROC, receiver operating characteristic; AUC, area under the curve.

To validate the identified key biomarkers and develop a more parsimonious model, we constructed a second ROC using only the top 4 most important features 02PAF, T3PAF, T4PAF and T5PAF (Figure 6).

We proceeded to conduct ROC curve analyses to investigate whether the two models might facilitate discrimination between PSCI and PSN (Figure 6). The logistic regression model and the Random Forest classifier demonstrated comparable and excellent discriminatory power, with AUCs of 0.761 and 0.773, respectively. In summary, both traditional logistic regression and Random Forest approaches identified a parsimonious set of EEG-based predictors, predominantly involving PAF in the temporal and occipital regions, that robustly discriminate patients with PSCI from healthy controls. The models demonstrated excellent discrimination, good calibration, and potential clinical utility.

In addition, we endeavored to incorporate all significant indicators for ROC curve analysis and uncovered an intriguing phenomenon. Across nearly all leads, the cutoff value for PAF was consistently found to be 8.9 Hz. This uniform cutoff value implies that, irrespective of the functional differences among brain regions, a reduction in PAF below 8.9 Hz may represent a common characteristic of PSCI. This finding reflects widespread neural oscillation abnormalities and suggests that the overall suppression of the α band could be a fundamental mechanism underlying PSCI (Supplementary Table 2).

## Discussion

In recent years, the assessment of cognitive function using peak alpha frequency (PAF) and alpha PSD has emerged as a prominent area of research, garnering significant attention (Clements et al., 2021; Mahjoory et al., 2019). Numerous studies have established a positive correlation between alpha activity and cognitive function (Seleznov et al., 2019; Williams Roberson et al., 2022). While previous studies have suggested PAF as an index for cognitive ability in a large variety of performance measures, but there always remains few studies in Poststroke Cognitive Impairment (PSCI). There are numerous factors influencing the current scale assessment, including the cognitive level of subjects, limb dysfunction, comprehension impairment, and others. Additionally, there remains a lack of objective assessment methods. However, our EEG index can serve as an objective supplementary tool for evaluating the cognitive function of individuals with PSCI.

### Characteristics of alpha activity in PSCI patients

Numerous studies have established a correlation between slowed resting-state α activity and deterioration in attention and memory functions (Babiloni et al., 2006, 2007; Doppelmayr et al., 2005; Grabner et al., 2004; Schumacher et al., 2020).

In our study, we investigated differences in alpha activity among HC, PSN, and PSCI. The results revealed a significant reduction in PAF among PSCI patients compared to HC participants. This slowing of alpha rhythms has been consistently linked to cognitive decline, serving as a potential biomarker for conditions such as Alzheimer’s disease and autism spectrum disorder (López et al., 2020; López-Sanz et al., 2016).

The underlying mechanisms may involve stroke-induced β-amyloid (Aβ) deposition and microglial activation, which can disrupt the excitatory-inhibitory balance of cortical networks, thereby suppressing the generation of α rhythms (Kang et al., 2023).

At the circuit level, thalamocortical dysfunction represents another key mechanism. The thalamus, particularly the thalamic reticular nucleus, serves as a pacemaker for alpha oscillations, and cerebrovascular injury may disrupt these critical regulatory circuits. This thalamocortical dysregulation could explain the widespread nature of alpha slowing observed in our PSCI patients (Hughes and Crunelli, 2005).

Furthermore, from a systems perspective, slower alpha peaks may reflect reduced neural metabolic efficiency and compromised information processing capacity. According to the neural efficiency hypothesis, optimal cognitive performance relies on efficient neural resource allocation, and alpha frequency slowing may indicate that compromised networks require more temporal cycles to process information (Ociepka et al., 2022). This is consistent with our findings that PAF varied with cognitive performance, with PSCI patients exhibiting significantly lower PAF than the PSN group.

The regional specificity of these effects, particularly in frontotemporal areas, suggests additional vulnerability in networks supporting higher-order cognition. Cortical thinning in prefrontal and temporal areas reflects a loss of neurons and synapses and is associated with functional decline (Molad et al., 2019). While alterations in cerebral blood flow and metabolism within these regions further contribute to cognitive impairment. Petrovic et al. (2017) similarly identified slowed alpha generation and synchronization as potential biomarkers of post-stroke cognitive impairment and compensatory reorganization.

Overall, our results support the view that PAF slowing, particularly in frontotemporal regions, may serve as an electrophysiological signature of post-stroke cognitive impairment, reflecting disruptions across multiple levels of neural organization–from molecular and cellular mechanisms to circuit-level dynamics and systems-level efficiency. These findings position alpha frequency as a sensitive indicator of the neurostructural and neurovascular disruptions underlying PSCI.

### Relationships between alpha activity and cognition in PSCI patients

Across the entire cohort, MoCA scores showed a positive correlation with PAF values at almost all electrodes; however, after false discovery rate (FDR) correction, only the correlation with PAF at the T3 and T4 electrode remained statistically significant. This suggests that although alpha rhythm may broadly relate to cognitive function, more robust and specific associations are localized to particular regions. First, the cognitive role of alpha activity in the temporal lobe has been well established. Using MEG, Foster et al. (2017) demonstrated that alpha activity in the temporal cortex plays an active inhibitory role in working memory, rather than merely reflecting passive processes. This aligns with our finding that T3/T4 activity is associated with abstract thinking, both in anatomical location and cognitive function. Second, the central role of the right temporoparietal network in executive function is widely recognized. Bagherzadeh et al. (2020) reported that alpha oscillations in the parietal and temporal lobes serve as sensitive biomarkers of executive function. Benwell et al. (2019) emphasized the right-lateralized advantage in attentional control processes. Furthermore, EEG studies have also shown that alpha activity in temporoparietal regions is closely related to cognitive processes such as attentional reorienting (Sauseng et al., 2005). Therefore, we postulate that the PAF changes captured by the T4 electrode may genuinely reflect the functional status of the impaired right ventral attention-executive control network following stroke.

Our study revealed that in the PSCI patient cohort, the PAF values in brain regions including the right prefrontal cortex (F4, F8, FP2), temporal lobe (T4, T6), central area (C4), occipital lobe (O2), and midline region (Fz) were significantly negatively correlated with the MoCA abstraction subscore. This finding contrasts with the patterns observed in both the healthy control group and the PSN group, potentially untangling unique neuropathophysiological mechanisms in PSCI. This inverse relationship may reflect compensatory neural mechanisms or pathological slowing in networks supporting high-level cognitive processing after stroke (Keser et al., 2022; Liu et al., 2017). Compensatory hyperactivation and functional reorganization in the right hemisphere may also contribute (Han et al., 2024). Abstraction function is inherently largely dependent on right-hemisphere networks (Ueda et al., 2025). After stroke, homologous regions in the right hemisphere may be over-recruited in an attempt to compensate for functional deficits in the left hemisphere or other impaired brain regions (Dai et al., 2025).

Notably, the temporal regions appear to be critically involved. The sustained correlation at T3 and T4 after multiple comparisons correction aligns with the known role of the middle temporal region in memory and integrative cognitive functions (Goyal et al., 2018). These findings are supported by existing literature indicating that stroke-induced cognitive impairment disrupts normal alpha oscillatory activity (Sun et al., 2021), particularly in posterior and temporal cortices, which are essential for maintaining cognitive network integrity.

### The suitability of alpha activity for the diagnosis of PSCI

Our analysis employed a dual statistical approach–binary logistic regression and Random Forest–to identify the most sensitive EEG biomarkers for discriminating between PSCI and HC. Binary logistic regression analysis revealed that T4PAF (peak alpha frequency at the T4 electrode) was the single most discriminative predictor for distinguishing PSCI from HC. The Random Forest classifier, robust to multicollinearity and capable of capturing complex interactions (Becker et al., 2023), identified a set of four predictors with the highest importance scores for classification: O2PAF, T3PAF, T4PAF and T5PAF. The concurrence of T4PAF across both models underscores the particular vulnerability of the temporal lobes in PSCI. The inclusion of O2PAF (right occipital) by the Random Forest model suggests that PSCI might involve a broader network disruption beyond the temporal region, encompassing occipital visuospatial processing areas (Bonkhoff et al., 2020).

The selected alpha activity indicators demonstrated robust performance in screening for dichotomized PSCI. The logistic regression model and the Random Forest classifier achieved AUCs of 0.761 and 0.773, respectively, indicating good discriminatory power. Notably, the predictive performance of the T4PAF individual indicator was also optimal on its own. This reinforces the notion that peak alpha frequency in the temporal lobe may be a particularly strong indicator of cognitive status in PSCI. These results were consistent whether cognitive outcome was treated as dichotomous (PSCI vs. HC) or as numerical data from cognitive tests.

A previous study observing the peak frequency changes from mild cognitive impairment to Alzheimer’s disease indicated that when the posterior dominant alpha frequency falls below 9 Hz (the typical lower limit of the alpha band), the risk of conversion significantly increases (López et al., 2020). Our findings, showing a moderate level of AUC values, again confirm the potential of resting-state EEG biomarkers to serve as a valuable supplement substitute to the MoCA for post-stroke cognitive screening. It is important to note that due to the limited spatial resolution of scalp EEG, the observed associations between PAF at specific electrodes and cognitive performance should be interpreted as reflecting the general involvement of broader brain regions rather than pinpointing exact neural generators.

Our study has several limitations. First, we used MoCA to measure global cognitive function. However, the MoCA does not assess specific cognitive domains. As a result, its diagnostic accuracy may be limited. In future studies, we plan to include more targeted scales for evaluation. Additionally, all participants were recruited from a single institution in China. Cultural and educational factors are known to influence performance on cognitive screening tools like the MoCA. Therefore, the generalizability of our proposed biomarkers to other populations with different demographic and cultural backgrounds requires further investigation. Our study developed a Random Forest model that showed promising discriminative ability (AUC = 0.818) on an independent test set for classifying stroke patients. However, we acknowledge the limitations raised by the internal cross-validation. The considerable variability in the cross-validation AUC scores (mean: 0.689 ± 0.230) suggests that the model’s performance is not yet fully stable, likely due to the constrained sample size of our cohort. This underscores a risk of overfitting and highlights that our findings should be interpreted as preliminary and hypothesis-generating. The improvement in AUC on the independent test set is an encouraging sign of generalizability, but it may also be influenced by the specific distribution of the small test sample. Therefore, external validation in a larger, prospective cohort is essential to confirm the robustness and clinical utility of our model before any potential clinical application.

Electroencephalography measures may provide types of information different from those offered by neuropsychological scales, particularly in patients who cannot be adequately assessed using standard cognitive screening tools such as the MoCA or more comprehensive batteries. This includes individuals with stroke-related symptoms–such as apraxia, hemiplegia, or reduced alertness–as well as those non-fluent in the primary language of assessment. Even EEG setups with a limited number of electrodes can yield valuable physiological insights that complement behavioral measures, offering an alternative source of functional data where traditional testing is infeasible.

## Conclusion

In conclusion, the present findings reveal a clear association between PAF in EEG and cognitive function. These results strongly imply that α peak activity could be a key factor in evaluating cognitive abilities.

## Data Availability

The raw data supporting the conclusions of this article will be made available by the authors, without undue reservation.
