# Supplementary material for: Peak alpha frequency as an objective biomarker for cognitive assessment in post-stroke cognitive impairment
Source: Front Aging Neurosci. 2025 Oct 23;17:1639970. doi: 10.3389/fnagi.2025.1639970 (PMC12589030; doi:10.3389/fnagi.2025.1639970)
Supplement: Supplementary file 1 [file Table_1.docx]

**Supplementary table1**

| **Comparison of PSCI, PSN and HC in PAF** | | | | | | | |
| --- | --- | --- | --- | --- | --- | --- | --- |
| Region | PSCI(n=41) | PSN(n=24) | HC(n=38) | *H(df)* | *P-value* | *ε²* | *Post-hoc comparisons* |
| FP1 | 8.5(7.1, 10.3) | 9.15(7.6, 11.5) | 9.3(8.1, 10.7) | 10.67(2) | 0.005 | 0.10 | PSN, HC > PSCI |
| FP2 | 8.5(7.1, 10.5) | 9.0(7.1, 10.0) | 9.3(7.6, 10.7) | 8.48(2) | 0.014 | 0.08 | HC > PSCI |
| F3 | 8.5(7.1, 10.5) | 9.15(7.6, 10.0) | 9.3(8.1, 10.7) | 10.29(2) | 0.006 | 0.10 | PSN, HC > PSCI |
| F4 | 8.5(7.1, 10.5) | 9.15(7.3, 10.0) | 9.15(8.73,10.0) | 10.45(2) | 0.05 | 0.10 | PSN, HC > PSCI |
| C3 | 8.8(7.1, 11.7) | 9.15(7.3, 10.7) | 9.3(8.1, 13.7) | 9.96(2) | 0.07 | 0.10 | HC > PSCI |
| C4 | 8.8(7.1, 12.0) | 9.3(7.6, 10.7) | 9.5(8.1, 12.7) | 16.04(2) | 0.0003 | 0.17 | HC > PSCI |
| P3 | 8.8(7.1, 12.0) | 9.5(7.1, 11.0) | 9.8(7.6, 12.7) | 9.77(2) | 0.008 | 0.10 | HC > PSCI |
| P4 | 9.0(7.1, 12.0) | 9.3(7.3, 11.5) | 9.65(8.1, 12.7) | 9.47(2) | 0.009 | 0.09 | HC > PSCI |
| O1 | 9.0(7.1, 12.0) | 9.3(8.1, 11.5) | 9.5(7.3, 12.0) | 7.44(2) | 0.031 | 0.07 | HC > PSCI |
| O2 | 8.8(7.1, 10.5) | 9.15(7.3, 11.5) | 9.5(7.3, 12.7) | 10.84(2) | 0.004 | 0.11 | HC > PSCI |
| F7 | 8.5(7.1, 10.5) | 9.3(7.1, 10.3) | 9.3(7.6, 10.7) | 11.90(2) | 0.003 | 0.12 | PSN, HC > PSCI |
| F8 | 8.5(7.1, 12.9) | 9.0(7.1, 10.3) | 9.4(8.1, 12.0) | 8.99(2) | 0.011 | 0.09 | HC > PSCI |
| T3 | 8.5(7.1, 12.5) | 9.15(7.6, 10.5) | 9.5(8.1, 11.7) | 13.66(2) | 0.001 | 0.13 | PSN, HC > PSCI |
| T4 | 8.5(7.1, 12.0) | 9.3(7.1, 11.5) | 9.65(7.8, 11.7) | 16.84(2) | 0.0002 | 0.17 | HC > PSCI |
| T5 | 8.8(7.1, 12.2) | 9.15(8.1, 11.0) | 9.65(7.6, 12.2) | 10.51(2) | 0.005 | 0.10 | HC > PSCI |
| T6 | 8.8(7.1, 10.7) | 9.4(7.1, 11.5) | 9.5(7.8, 12.2) | 12.95(2) | 0.002 | 0.13 | PSN, HC > PSCI |
| FZ | 8.5(7.1, 10.5) | 9.0(7.3, 10.0) | 9.3(8.1, 10.7) | 11.13(2) | 0.004 | 0.11 | PSN, HC > PSCI |
| CZ | 8.8(7.1, 11.2) | 9.15(7.3, 10.7) | 9.3(8.1, 12.7) | 10.80(2) | 0.005 | 0.11 | HC > PSCI |
| PZ | 9.0(7.1, 12.0) | 9.3(7.3, 11.0) | 9.5(8.1, 12.7) | 10.25(2) | 0.006 | 0.10 | HC > PSCI |
| Note: Data are presented as median. H: Kruskal-Wallis statistic; df: degrees of freedom; ε²: epsilon-squared effect size; n.s.: not significant. PSCI, Poststroke cognitive impairment; PSN, poststroke non-impaired; HC, healthy controls; PAF, peak alpha frequency. Post-hoc comparisons were performed using Dunn's test with Bonferroni adjustment.  Significant results (*P*<sub>adj</sub> < 0.05) are summarized in the final column. | | | | | | | |
|  |  |  |  |  |  |  |  |

**Supplementary table2**

| ROC curve thresholds and corresponding TPR/FPR Values for the PAF | | | | | | |
| --- | --- | --- | --- | --- | --- | --- |
| Region | AUC | Cut-off value | *P* | Sensitivity | Specificity | 95% CI |
| FP1 | 0.698 | 8.9 | 0.003 | 0.711 | 0.634 | 0.582, 0.814 |
| FP2 | 0.687 | 8.9 | 0.004 | 0.711 | 0.634 | 0.570, 0.805 |
| F3 | 0.698 | 8.65 | 0.002 | 0.789 | 0.512 | 0.582, 0.814 |
| F4 | 0.703 | 8.65 | 0.002 | 0.763 | 0.561 | 0.589, 0.817 |
| C3 | 0.7 | 8.9 | 0.002 | 0.763 | 0.561 | 0.585, 0.816 |
| C4 | 0.756 | 8.9 | ＜0.0001 | 0.789 | 0.61 | 0.650, 0.862 |
| P3 | 0.701 | 8.9 | 0.002 | 0.868 | 0.512 | 0.585, 0.816 |
| P4 | 0.697 | 9.9 | 0.003 | 0.447 | 0.854 | 0.582, 0.813 |
| O1 | 0.648 | 8.9 | 0.023 | 0.816 | 0.488 | 0.525, 0.772 |
| O2 | 0.716 | 8.9 | 0.001 | 0.789 | 0.512 | 0.604, 0.828 |
| F7 | 0.724 | 8.65 | 0.001 | 0.816 | 0.561 | 0.611, 0.843 |
| F8 | 0.695 | 8.9 | 0.003 | 0.711 | 0.634 | 0.656, 0.866 |
| T3 | 0.732 | 8.9 | ＜0.0001 | 0.816 | 0.585 | 0.587, 0.822 |
| T4 | 0.761 | 8.9 | ＜0.0001 | 0.737 | 0.659 | 0.626, 0.847 |
| T5 | 0.704 | 8.9 | 0.002 | 0.789 | 0.585 | 0.587, 0.822 |
| T6 | 0.737 | 9.15 | ＜0.0001 | 0.737 | 0.659 | 0.626, 0.847 |
| FZ | 0.713 | 8.9 | 0.001 | 0.711 | 0.659 | 0.599, 0.827 |
| CZ | 0.716 | 8.9 | 0.001 | 0.737 | 0.634 | 0.602, 0.830 |
| PZ | 0.712 | 8.9 | 0.002 | 0.842 | 0.463 | 0.591, 0.820 |
| Note. Detail data of ROC curves of EEG indices for the discrimination of PSCI and PSN group. AUC, areas under the curves; PAF, peak alpha frequency; 95% CI (Confidence Interval). | | | | | | |

**Supplementary figure1**

| 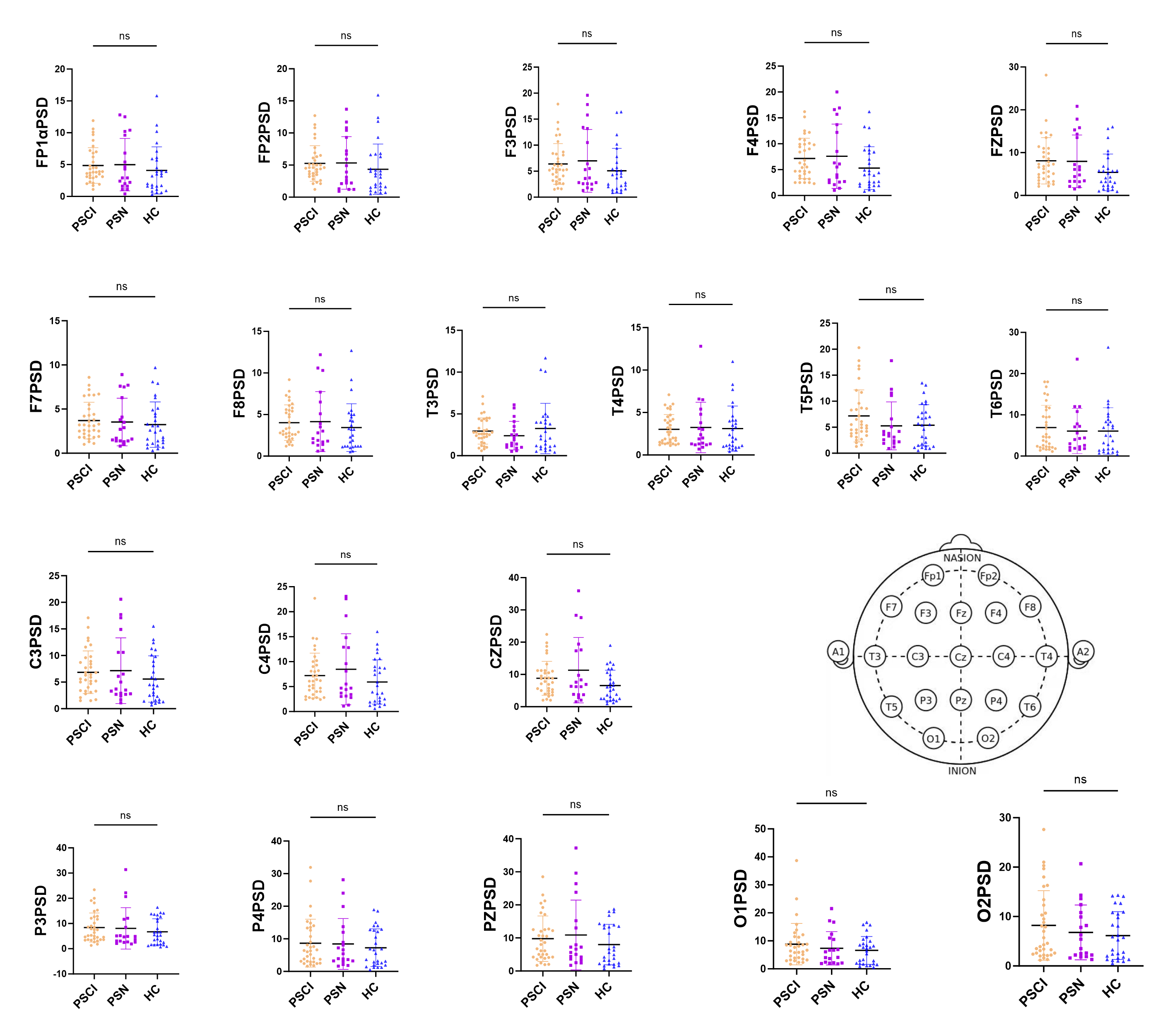 |
| --- |
| **Figure 1 Dot plots of the Alpha PSD among three groups**  Note. The alpha PSD showed no significant differences among the three groups across any of the leads. PSCI, Poststroke cognitive impairment; PSN, poststroke non-impaired; HC, healthy controls; PAF, peak alpha frequency.  *Represents *P* < 0.0167 after Bonferroni correction.  ns represents no statistical difference (*P* ≥ 0.0167). |

**Supplementary figure2**

| 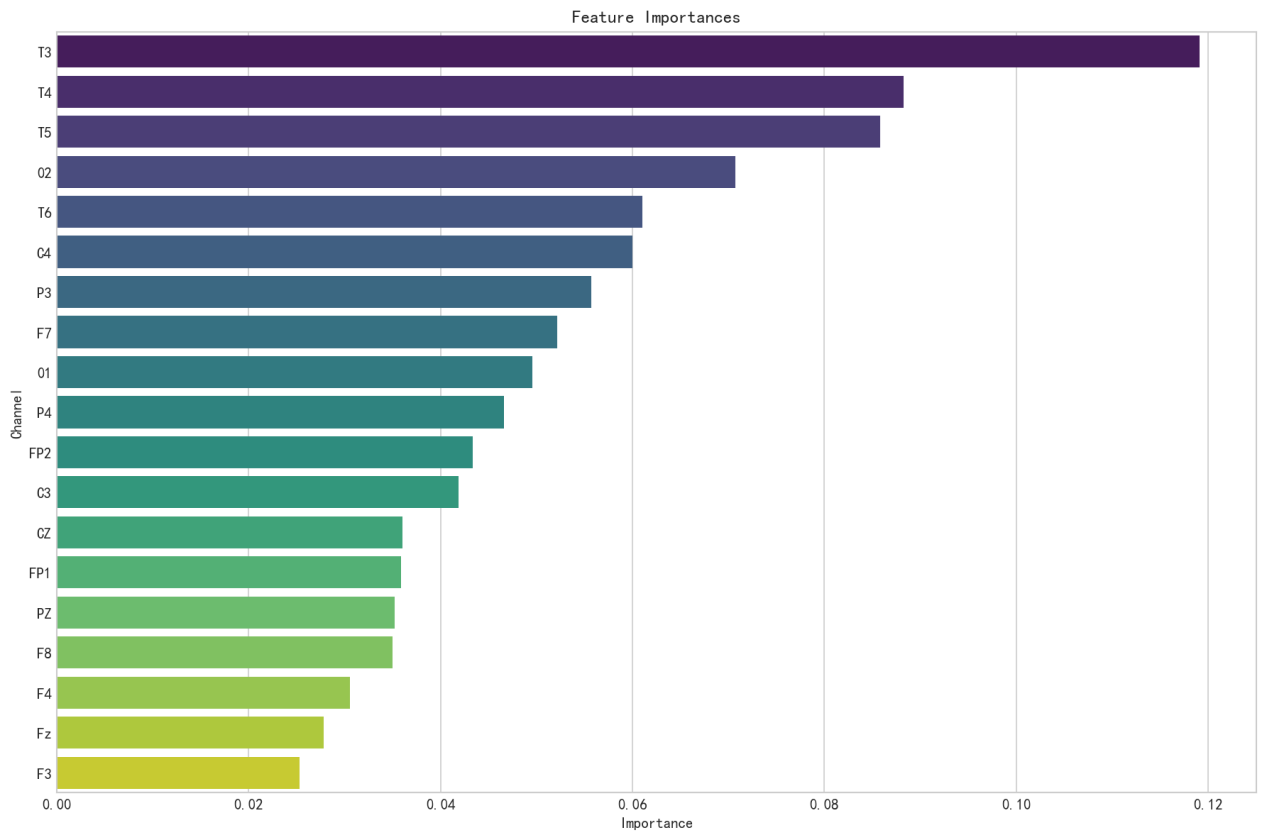 |
| --- |
| **Figure 2 Feature importance ranking derived from the machine learning model**  The graph illustrates the relative importance of the 19 features (EEG channels) in predicting PSCI. Features T3PAF, T4PAF, T5PAF, and O2PAF contributed the most to the model’s decision-making process. The importance values were calculated based on the mean decrease in Gini impurity from the Random Forest algorithm. |

**Supplementary figure 3**

| 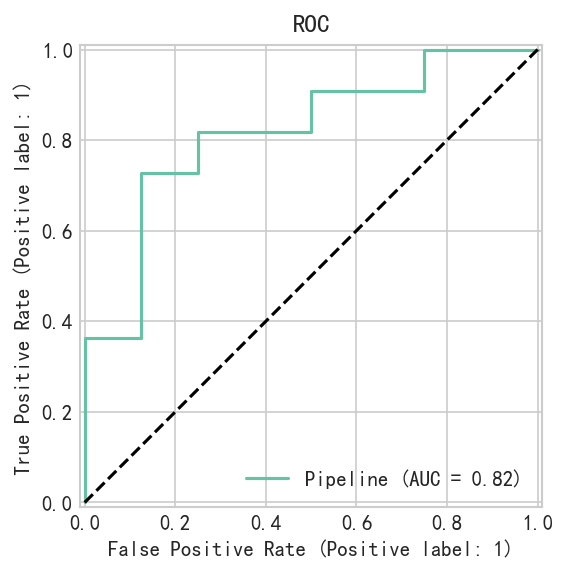 |
| --- |
| **Figure 3 Receiver operating characteristic (ROC) curves of the random forest classifier**  The solid green line represents the ROC curve of the model using all features, with an area under the curve (AUC) of 0.82. |

**Supplementary figure 4**

| 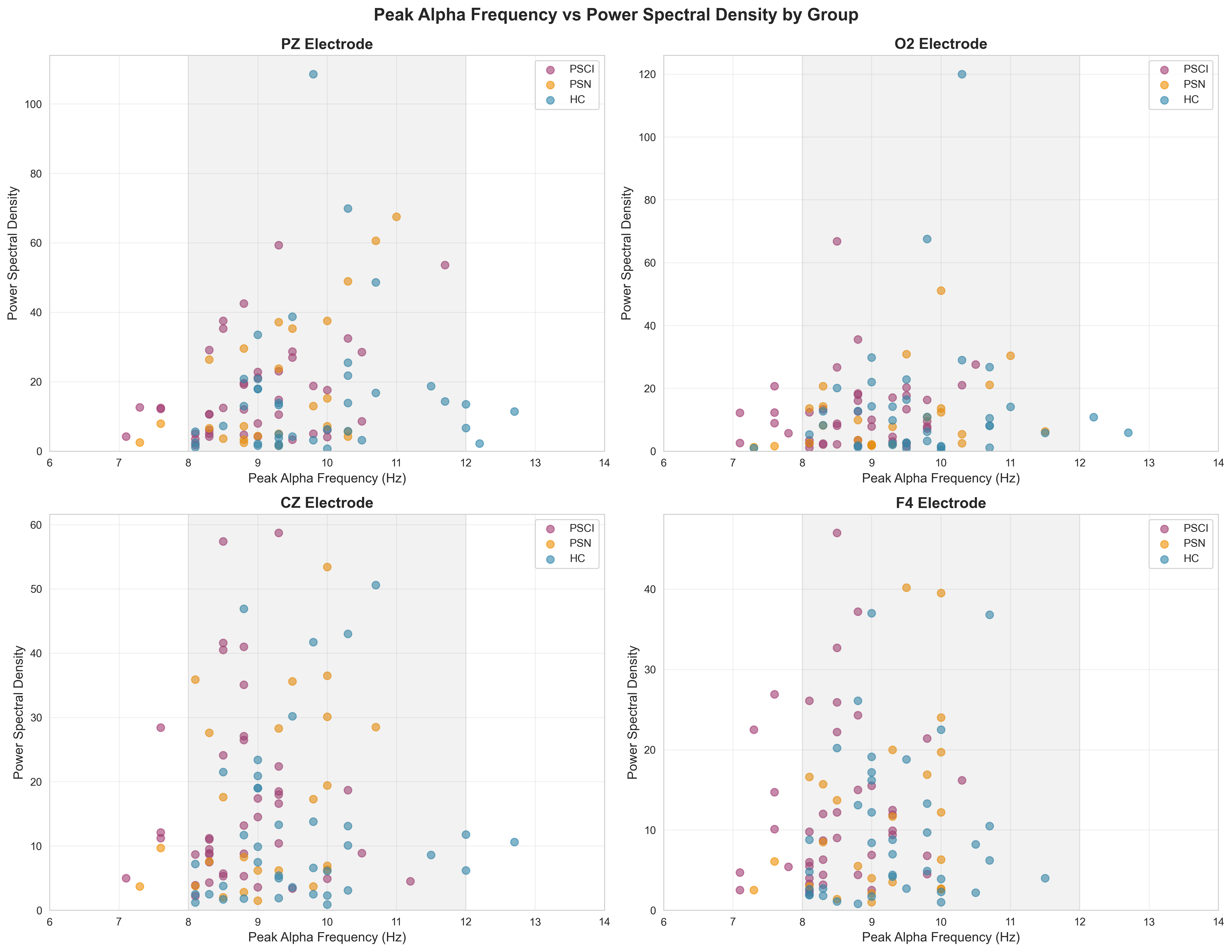 |
| --- |
| **Figure 4 Scatter plots of peak alpha frequency versus power spectral density for PSCI, PSN, and HC groups at four electrode locations**  Scatter plots illustrating the relationship between peak alpha frequency (PAF) and power spectral density (PSD) for the three experimental groups (PSCI, PSN, and HC) at four representative electrode locations: (A) PZ, (B) O2, (C) CZ, and (D) F4. The gray shaded area denotes the conventional alpha frequency band (8-12 Hz). onounced separation observed at posterior locations (PZ and O2). |
